# Antidepressant, anxiolytic, antipyretic, and thrombolytic profiling of methanol extract of the aerial part of *Piper nigrum*: In vivo, in vitro, and in silico approaches

**DOI:** 10.1002/fsn3.2047

**Published:** 2021-01-05

**Authors:** Nazim Uddin Emon, Safaet Alam, Sajib Rudra, Susmita Roy Riya, Avi Paul, S M Moazzem Hossen, Ummay Kulsum, Amlan Ganguly

**Affiliations:** ^1^ Department of Pharmacy International Islamic University Chittagong Chittagong Bangladesh; ^2^ Department of Pharmacy State University of Bangladesh Dhaka Bangladesh; ^3^ Department of Botany University Chittagong Chittagong Bangladesh; ^4^ Department of Pharmacy University of Dhaka Dhaka Bangladesh; ^5^ Department of Pharmacy Southern University Bangladesh Chittagong Bangladesh; ^6^ Department of Pharmacy University Chittagong Chittagong Bangladesh; ^7^ Department of Clinical Pharmacy and Pharmacology University of Dhaka Dhaka Bangladesh

**Keywords:** ADME/T, antidepressant, antipyretic, anxiolytic, molecular docking, *Piper nigrum*, thrombolytic

## Abstract

*Piper nigrum* L. also called black pepper is popular for its numerous uses. The present research is designed to investigate the pharmacological potential of methanol extract of *Piper nigrum* (MEPN). The antidepressant investigation was performed by using both in vivo forced swimming test (FST) and tail suspension test (TST) methods while the anxiolytic research by hole‐board test (HBT) method. Again, the antipyretic analysis was conducted through yeast‐induced pyrexia method, whereas clot lysis activity was employed by the thrombolytic method. Furthermore, *in silico* studies followed by molecular docking analysis of several secondary metabolites, pass prediction, and ADME/T were evaluated with AutoDock Vina, Discovery Studio 2020, UCSF Chimera software PASS online, and ADME/T online tools. The plant extract demonstrated dose‐dependent potentiality in antidepressant, anxiolytic, antipyretic, and thrombolytic activities. Induction of MEPN produced a significant (*p* < .5, *p* < .001) increase of mobility in FST and TST, and increased the head dipping and decreased the latency of time (*p* < .01, *p* < .001) in HBT. MEPN 400 (mg/kg; b.w.; p.o.) lowered the rectal temperature of yeast‐induced pyrexia substantially (*p* < .001). Besides, MEPN produced promising (*p* < .001) clot lysis activity. In the computational approach, among all the proteins, a docking score was found ranging from −1.0 to −7.90 kcal/mol. Besides, all the compounds were found safe in ADME/T study. The results of our scientific research validate the suitability of this plant as an alternative source of novel therapeutics.

## Graphical abstract

The methanolic extract of the plant Piper nigrum has promising biological activities that are supported by computational approaches. So, this plant can be considered as an alternative source of therapeutics.

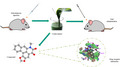

## 1 INTRODUCTION

Anxiety and depression are the most common causes of heterogeneous psychiatric illnesses in the world (Fajemiroye et al., 2016). Human neurological disorders have the potential to produce fear and dramatically impair normal activity and standard of living (Adnan et al., 2020). Depression can seriously disrupt the person and contribute to physical infirmity involving increased morbidity and mortality. However, anxiety is another prevalent psychiatric condition worldwide (Möller et al., 2016). Additionally, depressive disorders enlisted by the World Health Organization contribute substantially to worldwide nonfatal diseases (Depression, 2017). Anxiety, a natural emotional state, can be considered as a psychological disorder if occurs frequently. The manifestation of anxiety associated with depression contributes to multifaceted symptoms including declines in real prognosis, and deterioration in responsiveness to medication or therapy along with the increased risk of suicide (Kara et al., 2000).

Stress can characterize the pathogenesis of anxiety. Oxidative stress which has been identified as a causative factor contributing to the pathogenesis of a variety of chronic diseases including hepatic injury, diabetes, obesity, inflammation, neurological disorders, and cancer is also the product of the stressful condition (Hassan et al., 2014). Fever (pyrexia) is an example of body's complicated immune physiological response to infectious or inflammatory stimuli that induces a series of biochemical generating different endogenous pyrogens (Muhammad et al., 2019). Graft rejection, infection, malignant tumors, or other illnesses, and fever or hyperpyrexia may result in as a consequence of damaged tissue (Hossain et al., 2014). The high production of pro‐inflammatory mediators such as interleukin‐1β, interleukin‐α, interleukin‐β, and TNF‐α increase the synthesis of PGE2 (prostaglandin E2) near the preoptic hypothalamus zone and can force body temperature to rise (Rashed‐Al‐Qayum et al., 2013). While significant side effects such as gastric mucosal disruptions have spread to ulcerative states in extreme cases, there are many commercially available drug moieties to mitigate the effects of fever incidence (Debnath et al., 2013).

The interest of recent research is moved toward natural phytomedicines to discover novel herbal anti‐inflammatory compounds without adverse effects in order to combat these unwanted circumstances demonstrated by conventional drugs (Hossain et al., 2014). Again, blood clots (thrombosis) can contribute to coronary blood anomalies including acute myocardial infarction and brain hemorrhage which can lead to death (Emon et al., 2020). The first‐line medication for thrombus therapy is intravenous heparin because of its safety, potency, and viability (Prasad et al., 2006a). While streptokinase and urokinase are commonly prescribed due to their lower cost profile than other thrombolytic medications, they are also harmful as they can induce serious bleeding, re‐occlusion, and re‐infarction (Haines & Bussey, 1995). Computer simulated screening can well characterize the pharmacological functioning of phytochemicals (Parasuraman, 2011). To ascertain the design and preparation of new drug molecules using computer‐aided drug discovery techniques, the application of molecular docking is also important. The native ligand can recognize the binding site of the three‐dimensional protein structure and correlate with physiochemical interactions through successful molecular docking (Guedes et al., 2014).

In the treatment of chronic diseases, including moderate fever and life‐threatening diseases, medicinal plants are commonly used around the world (Adnan et al., 2019). They are also an noteworthy source of medicine in the urban areas of Bangladesh and demonstrate a pivotal role in the treatment of various disease states (Mollik et al., 2010). The traditional folkloric practice of medicinal plants relies primarily on scientifically sound experimental evidence. The Piperaceae family offers many secondary metabolites with high potential for medicinal use that are biologically active. Many species of *Piper* have a broad source of natural compounds including alkaloids and amides (Scott et al., 2005). Docking methodology is a standard computational tool in drug design to optimize major compounds and find novel active molecules via virtual screening along with experimental binding modes and affinities of small molecules (Parenti & Rastelli, 2012). Search algorithms are used to investigate the free energy landscape and to find out the best ligand poses in molecular docking (Huang & Caflisch, 2011). Usually, structure‐based strategy employing ligand − receptor molecular docking is adopted to predict interaction affinities and binding modes of the biological molecules with a particular target receptor (Guedes et al., 2014).

*Piper nigrum* (Family: Piperaceae) is also referred to be black pepper and is harvested for its fruits. The dried fruits of this plant have been consumed as a spice throughout the world. The plant is a permanent climber that holds to the supporting tree. The sedate small white flowers have appeared in thick pendulous spike. The pepper seeds have a diameter of approximately 0.5~1.0 cm. At maturity, they become yellowish‐red and carry only one seed in each. The length of the spike varies greatly with the cultivar. Young fruits are whitish‐green, green, or light purple, while mature fruits seem to be deep purple or deep yellow, and become red when peak (Saha et al., 2013). *P. nigrum*, having biological properties is well‐known as CNS stimulant, antifeedant, painkillers, and antipyretics (Miyakado et al., 1980). *P. nigrum* is found to comprehend bioactive compounds such as “betaine, achyranthine, 10‐tricosanone, 10‐octacosanone, 4‐tritriacontanone, piperolactam D, pellitorine, piperidine, piperine, sylvamide, cepharadione A, and paprazine” (Ee et al., 2009). Though *P. nigrum* has many significant drug characteristics, no study regarding the quest of antidepressant, anti‐anxiolytic, antipyretic, and thrombolytic activity of the aerial part of this plant has been carried out till today to the best of our knowledge. For that reason, the neuropharmacological, antipyretic, and thrombolytic effectiveness of the methanol extract of the aerial part of *Piper* *nigrum* has been aimed to conduct through biological (in vivo and in vitro) and computational (in silico) methodologies.

## 2 MATERIALS AND METHODS

### 2.1 Collection and identification of the plants

In September 2019, *Piper nigrum* were collected from Rangamati, Chittagong, Bangladesh. After that taxonomy of the plant was identified by the taxonomist namely Sajib Rudra, Department of Botany, University of Chittagong, Chittagong‐4331, Bangladesh. After that, the plant was stored for further analysis (Accession No: CTGUH SR‐1780). The aerial part of the plant was segregated after washing, and plant materials were dried for twenty days in a shaded place at low temperatures and ground using appropriate blender equipment. The material was then placed in an impermeable package and held in a cool, dry, and warm position until further inspection.

### 2.2 Extraction process

The powdered content (approximately 800 g) was stored in a glass bottle and soaked in 2.4 L of methanol. This was then set and sustained for 15 days for the most intensive absorption with regular shaking and mixing. The solutions were filtered by the Whatman filter paper (Bibby RE200; Sterilin Ltd) after 15 days and condensed by subjecting to a water bath. Afterward, from the powdered materials, 7.2 g of crude extracts was developed followed by storage of concentrate for both the reservation and for the investigation at 4°C.

### 2.3 Animals and ethical consideration

To conduct the biological activity assessment, Swiss albino mice of 25–30 g weight were obtained from Jahangirnagar University. Prior to the testing, all animals were kept in the new environment for one week to get used to. The animals were housed in a well‐ventilated animal house during the experimental process at 25°C temperature, comparative humidity (55%–65%), and normal 12‐hr day/light pattern. The experimental animal models were provided with normal laboratory food and ad libitum drinking water.

### 2.4 Drug and chemicals

Commercially offered lyophilized streptokinase (SK) vials (Incepta Pharmaceuticals Ltd), paracetamol (Beximco Pharmaceuticals Ltd), methanol and Tween‐80 (Sigma‐Aldrin), and saline water (ACME Laboratories Ltd) had been purchased from the respective suppliers.

### 2.5 Acute toxicity test

The research was carried out under normal laboratory conditions, directed by Organization for Environmental Control Development (Viran et al., 2003). Every group of 10 mice were administered oral doses of MEPN 1,000, 2,000, and 3,000 mg/kg, whereas the control group received only vehicle (water). For 48 hr, the groups were tracked. In addition, the animals were weighed and physical, cognitive, and/or any deleterious impact on animal models were documented every day.

### 2.6 Forced swimming test (FST) on mice

The forced swimming test was performed to evaluate the antidepressant effect of MEPN in mice according to the method formerly mentioned (Cryan et al., 2005). Introductory research was performed the day before the final study, to adapt the animal models to the experimental system. The investigational swimming equipment was made up of a transparent glass tank (25 ± 15 ± 25 cm) filled with water up to 15 cm (25 ± 1°C) to facilitate swimming. The mice were divided into five groups, with six mice in each group. Group I was given the vehicle, whereas Group II was treated with standard medication (diazepam: 5 mg/kg, b.w.; i.p.) and Groups III–V received the test sample (MEPN 100, 200, and 400 mg/kg, b.w.; p.o.). After thirty minutes, each mouse was put in the tank for 6 min, where the initial adjustment period was considered to be the first couple of minutes and the immobility period was reported for succeeding 4 min.

### 2.7 Tail suspension test (TST) on mice

A simple and efficient way to measure the antidepressants’ action is the tail suspension test (Wang et al., 2019). Mice are classified into five classes, each of which consists of six mice. Group I was given the vehicle, whereas Group II was treated with standard medication (diazepam: 5 mg/kg, b.w.; i.p.) and Groups III–V received the test sample (MEPN 100, 200, and 400 mg/kg, b.w.; p.o.). After the implementation of the test samples, the mice were subjected to a depressed state (immobility), which was kept at the end of the tail with adhesive tape (nearly 1 cm from the tip of the tail). Only the last four minutes of the immobility period were reported for each mouse in all groups, for six minutes of examination in total.

### 2.8 Hole‐board test (HBT) on mice

The hole‐board test was performed following the previously described method by Moniruzzaman et al. (2015). For the analysis, a hole‐board apparatus was constructed which is consisted of a wooden box distributed uniformly (40 × 40 cm) with 16 holes (each 3 cm in diameter). The unit was raised to a height of 25 cm. The mice were divided into five groups, with six mice in each group. Group I was given the vehicle, whereas Group II was treated with standard medication (diazepam: 5 mg/kg, b.w.; i.p.) and Groups III–V received the test sample (MEPN 100, 200, and 400 mg/kg, b.w.; p.o.). After 30 min of administration, the mice were placed into the device. Head dips were documented for more for five minutes. As a standard anxiolytic agent, diazepam (5 mg/kg, i.p.) was introduced.

### 2.9 Yeast‐induced pyrexia on mice

Brewer's yeast‐induced pyrexia approach was applied to mice models with the subcutaneous injection of yeast suspension ensuring a dosage of 10 ml/kg body weight to determine the antipyretic function of the methanol extract of *P. nigrum* (Loux et al., 1972). In this study, animal models were limited from food intake overnight prior to the experimentation, despite allowing access to unlimited water. Preliminary rectal temperatures were documented using an Ellab thermometer (Hossain et al., 2014). Mice were only chosen for the antipyretic property evaluation if the rectal temperature increased by 0.3 to 0.5°C after 18 hr of subcutaneous injection of yeast solution. *P. nigrum* extracts were administered orally at three distinct doses (100, 200, and 400 mg/kg body weight) along with paracetamol (100 mg/kg body weight orally) as the standard drug, whereas only distilled water (10 ml/kg) was obtained by the control group. To conduct the antipyretic activity assessment, mice were selected only when there is an increase in rectal temperature of 0.3–0.5°C after 18 hr of subcutaneous administration of yeast solution (20%). Extracts of *P. nigrum* were introduced orally at three different doses (100, 200, and 400 mg/kg body weight) along with paracetamol (100 mg/kg body weight orally) as reference drug where control group received distilled water (10 ml/kg) only. Finally, rectal temperature tracking lasted for 3 hr with an interval of 1 hr (Abena et al., 2003).

### 2.10 In vitro thrombolytic study

#### 2.10.1 Streptokinase (SK) solution preparation

To the commercially available SK‐1500000 I.U. (Polamin‐Werk GmbH), 5 ml of pure distilled water was added and mixed properly. 100 μl (30,000 I.U) from the suspension was taken as a stock solution for the in vitro thrombolysis investigation (Emon et al., 2007).

#### 2.10.2 Specimen for thrombolytic test

With minor adjustment, the experiment was conducted using the previously developed protocol (Emon et al., 2006b). Blood (5 ml) from physically strong human volunteers (*n* = 10) with no records of NSAID and anticoagulant consumption for previous 10 days was considered for this study. 500 μl of blood was taken to the early Eppendorf measurement and stored for 45 min at 37°C in the incubator. The serum was finally isolated from Eppendorf following the formation of coagulation. In order to determine the exact weight of coagulation, each tube containing only coagulation has been further weighed. After that, 100 μl of plant extract was introduced to the tubes. In the incubator, the test tube carrying plant extract was suspended and the temperature was set for 90 min at 37°C.

After the clot lysis, the blood serum was removed and the tube was again weighed to monitor the variation of weight followed by the development of the clot lysis. The clot lysis percentage was computed by applying the following formula:

$$\%\;\text{clot}\;\text{lysis}=\frac{\text{Weight}\;\text{of}\;\text{the}\;\text{lysis}\;\text{clot}}{\text{Weight}\;\text{of}\;\text{clot}\;\text{before}\;\text{lysis}}\times 10$$

### 2.11 In silico study

#### 2.11.1 Molecular docking: protein preparation

Human serotonin transporter (PDB ID: 5I6X; Coleman et al., 2016), potassium channel enzyme (PDB ID: 4UUJ; Lenaeus et al., 2014), microsomal prostaglandin E synthase 1 (PDB ID: 4YK5; Luz et al., 2015), and tissue plasminogen activator (PDB ID: 1A5H; Renatus et al., 1997) have been derived from RCSB Protein Data Bank (https://www.rcsb.org/structure) in PDB format for the antidepressant, anxiolytic, antipyretic, and thrombolytic studies, respectively. With Discovery Studio 2020, all the water and heteroatom have been eliminated from proteins. The nonpolar hydrogens and the Gasteiger charge were kept default to prepare proteins. Furthermore, all proteins were brought to minimum energy level throughout UCSF Chimera and processed for further analysis, using normal residues in AMBER ff14sB and other residues in Gasteiger mode (Shapovalov & Dunbrack, 2011).

#### 2.11.2 Molecular docking: ligand preparation

**FIGURE 1:**
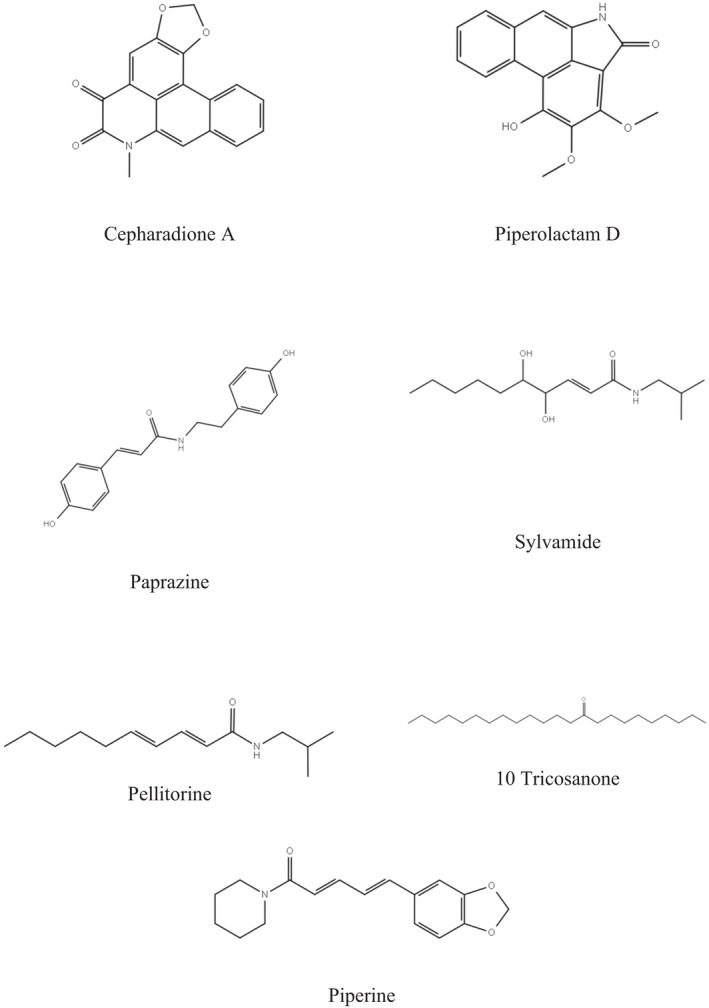
Chemical structures of cepharadione A, paprazine, pellitorine, piperine, piperolactam D, sylvamide, and 10‐tricosanone used in the computational study

The structure of seven compounds of *Piper nigrum* namely paprazine (PubChem CID: 5372945), pellitorine (PubChem CID: 5318516), piperine (PubChem CID: 638024), sylvamide (PubChem CID: 21580215), cepharadione A (PubChem CID: 94577), piperolactam D (PubChem CID: 14039008), and 10‐tricosanone (PubChem CID: 5195001) were retrieved from PubChem database (https://pubchem.ncbi.nlm.nih.gov/; Figure 1). Besides, diazepam (PubChem CID: 3016), ibuprofen (PubChem CID: 3672), and streptokinase (PubChem CID: 9815560) have been studied to compare and contrast the docking of the compounds of *Piper nigrum*. The ligands have been downloaded in 2DSDF format and minimized by PyRx tool aim to find the best possible hit for these targets. The virtual screening software PyRx from MGLTools (https://ccsb.scripps.edu/mgltools/) has been kept in default format (Herowati & Widodo, 2014).

#### 2.11.3 Molecular docking: docking analysis

For the docking of the chosen protein–ligand complexes, the PyRx AutoDock Vina has been used (Herowati & Widodo, 2014). A semiflexible docking system has been used for the docking analysis. PDB files of phytochemicals and proteins were minimized and subsequently transformed into PDBQT format using PyRx AutoDock Vina software. This analysis maintained the protein's rigidity and ligand's versatility. 10 degrees of freedom has been allowed to the ligand molecules. AutoDock specifies measures to transform the molecules into the pdbqt format molecules, box type, grid box formation, etc., automatically. The grid box was created with an active site in the center of the box. In addition, BIOVIA Discovery Studio Visualizer 2020 (Biovia, 2017) was accelerated to determine the best docking positions.

#### 2.11.4 Pharmacokinetics and toxicity measurement

The SwissADME online method has been used to assess the pharmacokinetic properties (ADME) of the compounds. Lipiniski's five rule (molecular weight not more than 500 dalton; H‐bond donors ≤5; H‐bond acceptors ≤10; molar refractivity ranging from 40 to 130; and lipophilicity <5) has been considered for the evaluation of positive drug‐like properties of any compound (Lipinski et al., 1997). In addition, the online tool admetSAR (http://lmmd.ecust.edu.cn/admetsar2) was used to calculate the toxicological properties of all the compounds.

#### 2.11.5 Pass prediction

For the quest of the potential biological effectivities of the selected compounds, the pass prediction was reviewed with the use of the PASS online tools (http://www.pharmaexpert.ru/passonline/predict.php). The Pa and Pi values ranged from 0.000 to 1.000 (Mojumdar et al., 2016). The compounds have been considered biologically seem to potentials whenever its Pa values are higher than the Pi values. In comparison, Pa < 0.7 suggests high drug activities, 0.5 < Pa < 0.7 shows moderate therapeutic potentials and Pa < 0.5 shows poor pharmaceutical activity (Goel et al., 2011; Khurana et al., 2011).

### 2.12 Statistical analysis

The data were analyzed as mean ± standard error mean (*SEM*). A one‐way ANOVA followed by Dunnett's *t* test was used to conduct statistical judgments. These values were compared with the control group and declared statistically significant when *p* < .001, *p* < .01, and *p* < .05. The statistical analysis was conducted using the software namely GraphPad Prism (version 5.2).

## 3 RESULTS

### 3.1 Effect of MEPN on oral acute toxicity

In the acute oral toxicity study, no significant morphological modifications (eyes, nose, ears, and fur) have been observed to the rodent. The MEPN was stable at a single dose up to 3,000 mg/kg body weight dose. That is why MEPN (200 and 400 mg/kg, b.w.; p.o.) was chosen as the doses for the biological investigations.

### 3.2 Effects of MEPN on FST

**FIGURE 2:**
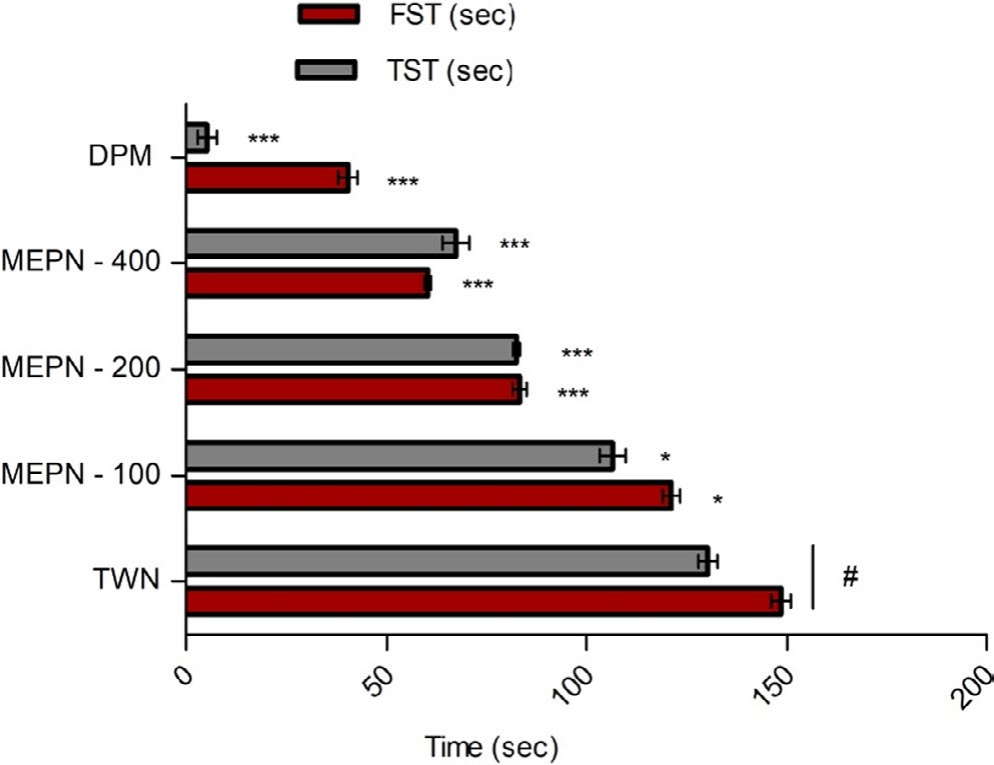
Effect of MEPN on the forced swimming test and tail suspension test. Data were presented as mean ± *SEM* (*n* = 6), and ∗*p* < .05, **p < .01, and ***p < .001 considered as significant. MEPN, methanol extract of the aerial part of *Piper nigrum*; STK, streptokinase; DPM, diazepam

The decreased immobility time (s) in the FST has been shown in Figure 2. With the increase in the dose, the immobility decreased in the dose‐dependent manner. The highest immobility estimates for MEPN 400 mg/kg; b.w.) and were 60.30 ± 0.78 (*p* < .001; s). In addition, standard drug (diazepam 5 mg/kg b.w.; i.p.) yielded the FST value of 40.36 ± 2.37 (*p* < .001; s).

### 3.3 Effects of MEPN on TST

In order to estimate the antidepressant activity, TST has been employed and the MEPN decreased the immobility time in the TST study in a dose‐dependent manner (*p* < .05, *p* < .001). The TST immobility times were 100.21 ± 1.28 (*p* < .05), 82.56 ± 0.90, and 67.30 ± 3.31 (*p* < 0,001; s) at the doses of 100, 200, and 400 mg/kg, b.w.; p.o.), respectively (Figure 2).

### 3.4 Effects of MEPN on HBT

**FIGURE 3:**
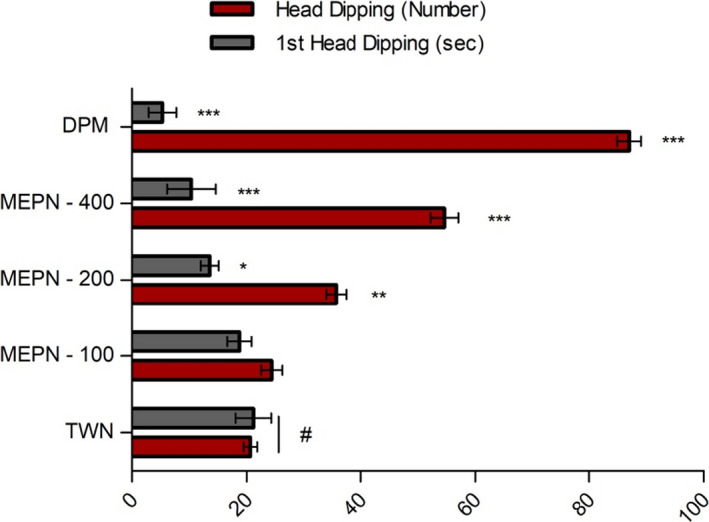
Effect of MEPN on the hole‐board test. Data were presented as mean ± *SEM* (*n* = 6), and ∗*p* < .05, *p < .01, and *p < .001 considered as significant. MEPN = methanol extract of the aerial part of *Piper nigrum*, DPM = Diazepam

The number of head dips did not increase substantially with the administration of MEPN 100 mg/kg/p.o. The initial head‐dip latency (s) and head poking was reduced substantially (*p* < .5, *p* < .01) by the increased dose. Sustainable (*p* < .01) explorative activity has been demonstrated by the reference drug (diazepam: 5 mg/kg, i.p.; Figure 3).

### 3.5 Effect of MEPN on yeast‐induced pyretic test

The rectal temperature was highly increased after 18 hr of Brewer's yeast suspension administration in the subcutaneous route. Paracetamol (100 mg/kg, i.p.)‐ and MEPN (100, 200, and 400 mg/kg)‐treated mice have shown a significant (*p* < .01, 0.001) dose‐dependent pyrexia inhibition compared with the control group. The maximum inhibition of pyrexia was produced by MEPN 400 mg/kg, which inhibition rate is close to the prescribed drug paracetamol‐100 mg/kg (Table 1).

**TABLE 1:** Brewer's yeast‐induced hyperpyrexia inhibition report of MEPN and control samples in mice model

Treatment	Dose (mg/kg b.w.; p.o.)	Normal rectal temperature (°F)	Rectal temperature (°F) after yeast administration	Rectal temperature (°F) after drug administration		
				60 min	120 min	180 min
TWN−80	10 ml/kg	98.13 ± 0.32	103.32 ± 0.86	102.90 ± 0.34^#^	102.33 ± 0.23^#^	102.88 ± 0.36^#^
PCL	100	98.50 ± 0.32	103.47 ± 0.33	99.14 ± 0.77***	98.88 ± 0.74***	97.82 ± 0.47***
MEPN	100	98.12 ± 0.88	103.77 ± 0.45	102.20 ± 0.66	102. 33 ± 0.47	102. 44 ± 23
MEPN	200	98.20 ± 0.45	104.04 ± 1.19	101.87 ± 0.47***	102.48 ± 0.38***	102.88 ± 1.15**
MEPN	400	97.24 ± 0.54	104.44 ± 0.72	100.88 ± 0.84***	101.34 ± 0.88***	101.90 ± 0.74***

Note

Effects of the MEPN on the hyperpyrexia inhibition test in mice (*n* = 6). Values are presented as mean ± *SEM*; one‐way analysis of variance (ANOVA) followed by Dunnett's test.

MEPN, methanol extract of the aerial part of *Piper nigrum*; PCL, paracetamol; TWN, 1% Tween‐80.

* *p* < .05, **p < .01, and ***p < .001 are considered as significant compared with the control, where # is designated as control.

### 3.6 Effect of MEPN on thrombolytic test

**FIGURE 4:**
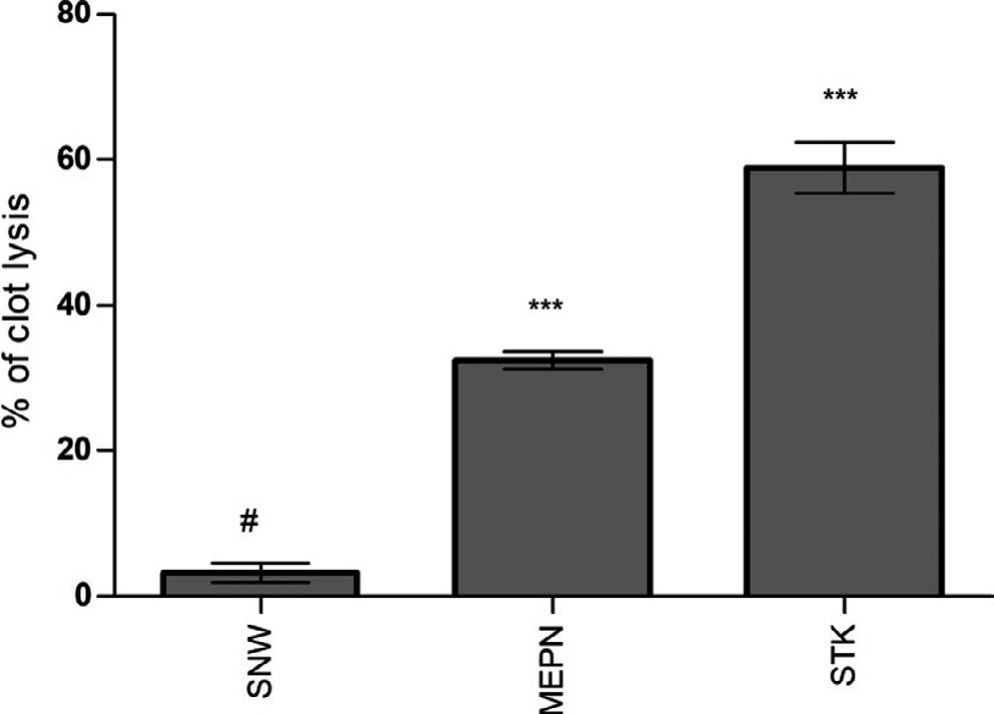
Effect of MEPN on the thrombolytic study (*n* = 10). Data were presented as mean ± *SEM* (*n* = 10), and ∗*p* < .05, *p < .01, and *p < .001 considered as significant. MEPN, methanol extract of the aerial part of the *Piper nigrum*; STK, streptokinase; SNW, saline water

A rise of 58.87 ± 3.52% (*p* < .001) of the coagulation with an hour and a half brooding at 37°C has been indicated the anticoagulant efficacy of 100 μl streptokinase (30,000 I.U.; a positive control anticoagulant). Furthermore, saline water displayed a therapeutically insignificant 3.23 ± 1.32% clot lysis. Figure 4 displays the anticlotting overview of the MEPN.

### 3.7 Molecular docking analysis for antidepressant and anxiolytic study

**FIGURE 5:**
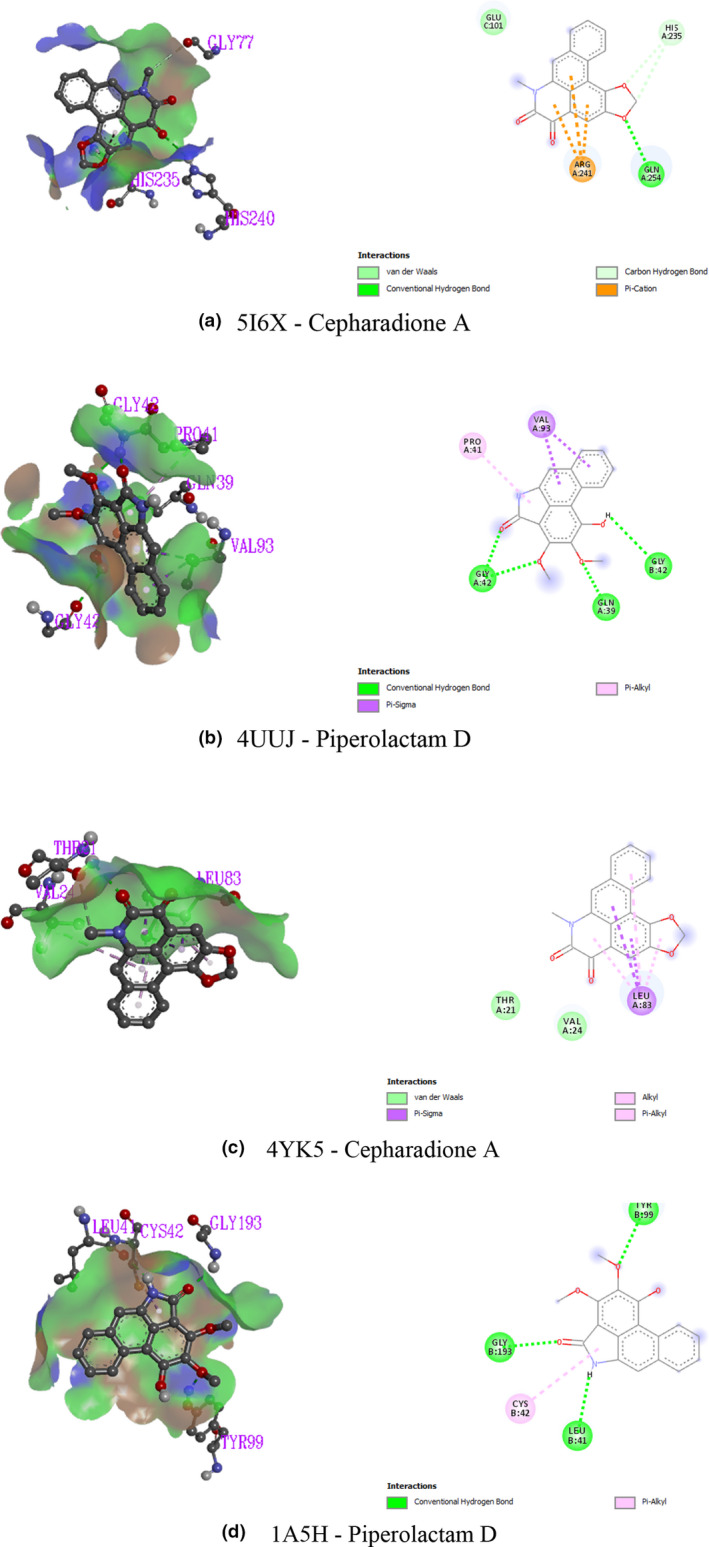
3D and 2D presentations of the best ligand–receptor interactions (a, b, c, and d represent 5I6X‐cepharadione A, 4UUJ‐piperolactam D, 4YK5‐cepharadione A, and 1A5H‐piperolactam D interactions, respectively)

The docking analysis results for antidepressant and anxiolytic activity are presented in Table 2 and Figure 5. In this investigation, two receptors namely human serotonin transporter (PDB ID: 5I6X) and potassium channel (PDB ID: 4UUJ) have been used to screen antidepressant and anxiolytic docking analysis, respectively. In the case of the human serotonin transporter (PDB ID: 5I6X), the ranking of the docking score is as follows: cepharadione A > piperolactam D > diazepam > paprazine > piperine > 10‐tricosanone > sylvamide > pellitorine. On the contrary, the docking of the selected compounds against the potassium channel (PDB ID: 4UUJ) is as follows: piperolactam D > diazepam >cepharadione A > piperine > paprazine > 10‐tricosanone > sylvamide > pellitorine. Amino acid residues namely arg241, gln254, his235, and glu101 established the interaction between cepharadione A and 5I6X. Furthermore, piperolactam D binds to the enzymatic pocket of 4UUJ receptor by means of gly42, gln39, gly42, pro41, and val93 residues with a docking score of −7.9 kcal/mol.

**TABLE 2:** Docking scores of the selected compounds with the human serotonin transporter (PDB ID: 5I6X), potassium channel (PDB ID: 4UUJ), mPGE‐1 (4YK5), and tissue plasminogen activator (PDB ID: 1A5H)

Docking score				
Compounds	Receptors			
	Antidepressant	Anxiolytic	Antipyretic	Thrombolytic
	5I6X	4UUJ	4YK5	1A5H
Cepharadione A	**−7.1**	−4.4	**−6.9**	−6.3
Paprazine	−5.6	−3.0	−6.3	−6.1
Pellitorine	−3.0	−1.0	−4.9	−2.1
Piperine	−4.6	−4.4	−6.2	−6.3
Piperolactam D	−6.6	**−7.9**	−4.5	−**7.2**
Sylvamide	−3.6	−2.0	−6.2	−2.9
10‐Tricosanone	−4.2	−2.8	−4.5	−3.1
Standard (Diazepam/paracetamol/streptokinase)	−6.4	−7.2	−5.4	−6.4

Note

The most significant values of docking scores have been marked in bold letter.

### 3.8 Molecular docking analysis for the pyretic study

The docking analysis of antipyretic activity has been illustrated in Table 2 and Figure 5. In this investigation, prostaglandin E synthase‐1 (4YK5) was used to quest of binding interaction with the selected compounds of *P. nigrum*. Cepharadione A explores best binding affinity with the prostaglandin E synthase‐1 (4YK5) proteins which interact with the 4YK5 receptor through the van der Waals (thr21, val24) and pi–sigma (leu83) bonds. The ranking of the docking score is as follows: cepharadione A > paprazine >piperine > sylvamide >paracetamol > pellitorine >piperolactam D > 10‐tricosanone.

### 3.9 Molecular docking analysis for thrombolytic study

The docking analysis results for the thrombolytic activity have been presented in Table 2 and Figure 5. In case of tissue plasminogen activator (PDB ID: 1A5H), the highest score has been obtained −7.21 kcal/mol for piperolactam D. The ranking of the docking score is as follows: piperolactam D > streptokinase > cepharadione A > piperine > paprazine > 10‐tricosanone > sylvamide > pellitorine. Piperolactam D interacts with the protein (1A5H) through the series of amino acid residues (leu41, cys42, gly193, tyr99) of conventional hydrogen and pi–alkyl bonds.

### 3.10 Pharmacokinetics and toxicity measurement

The study shows that all the compounds agree with Lipinski's rules and claim that these compounds are orally bioavailable. In order to predict the toxicological properties of the seven compounds, the online admetSAR (http://lmmd.ecust.edu.cn/admetsar2/) server is also used. The analysis showed that the selected compounds are non‐Ames toxic and noncarcinogenic (Table 3).

**TABLE 3:** Absorption, digestion, metabolism, excretion, and toxicological properties of the compounds for good oral bioavailability

Molecules	PID	MW (g/mol)	HBD	HBA	LogP (o/w)	GA	AMT	CAR
Cepharadione A	94577	305.3	0	4	2.26	High	No	No
Paprazine	5372945	283.32	3	3	2.05	High	No	No
Pellitorine	5318516	223.35	1	1	3.61	High	No	No
Piperine	638024	285.34	0	4	3.42	High	No	No
Piperolactam D	14039008	295.29	2	4	2.33	High	No	No
Sylvamide	21580215	257.37	3	3	3.14	High	No	No
10 Tricosanone	5195001	338.61	0	1	5.78	Low	No	No

Note

PID = PubChem ID, MW = molecular weight (acceptance range: <500), HBD = hydrogen‐bond donor (acceptance range: ≤5), HBA = hydrogen‐bond acceptor: (acceptance range: ≤10), LogP = high lipophilicity (acceptance range: <5), AMT, AMES toxicity; CAR = carcinogens; GA, gastro‐intestinal absorption.

### 3.11 PASS prediction study

Seven major selected compounds of *Piper nigrum* were studied by the PASS online tool for antidiarrheal and antibacterial activities, and Pa values higher than Pi showed strong molecular potency (Table 4).

**TABLE 4:** PASS prediction of standard drug and selected bioactive compounds of the aerial part of *Piper nigrum*

Biological Activity	Molecules													
	Cepharadione A		Paprazine		Pellitorine		Piperine		Piperolactam D		Sylvamide		10‐Tricosanone	
	Pa	Pi	Pa	Pi	Pa	Pi	Pa	Pi	Pa	Pi	Pa	Pi	Pa	Pi
Antidepressant	0.652	0.171	—	—	0.230	0.040	0.533	0.016	0.832	0.088	0.250	0.016	0.925	0.004
Anxiolytic	0.424	0.030	0.552	0.041	0.573	0.042	0.372	0.146	0.393	0.083	—	—	0.592	0.010
Antipyretic	0.045	0.025	0.264	0.027	0.481	0.042	—	—	—	—	0.496	0.039	0.656	0.019
Thrombolytic	—	—	0.601	0.072	0.622	0.007	0.172	0.115	0.713	0.116	0.050	0.045	0.713	0.018

## 4 DISCUSSION

Although multiple treatment approaches have been available to treat anxiety, depression, and coronary artery disease, it still remains a mystery to fully alleviate disease symptoms without side effects. Therefore, the clinical application of these drugs is constrained by side effects and poor pharmacokinetics. As a result, the safeties, effectiveness, onset of action, duration of action, and side effects of current drugs have become a critical issue and requirement of new drugs are becoming a major concern. Due to the diversity of neural targets, herbal medicine has become promising for the treatment of these diseases (Fajemiroye et al., 2016). Current research reveals that MEPN possesses noteworthy impact on force swimming, tail suspension, hole‐board head dipping, and anticoagulant property screening.

The forced swimming test (FST) and tail suspension test (TST) are popularly acknowledged techniques for antidepressant action estimation. For these studies, the characteristic behavior assessment, known as immobility, is considered to be like human depression. The capability to decrease immobility periods in mice is close to the drug's antidepressant potential (Porsolt et al., 1977). The adoption of swimming as an objective helped in identifying multiple potential compounds as antidepressants. Swimming event can be amended by BDNF (brain‐derived neurotrophic factor) and NT‐3 (neurotrophin‐3) antagonists, estradiol, kappa opioid receptor, glutamate metabotropic receptors, nitric oxide synthesis, melanocortin‐4, GABA (gamma‐aminobutyric acid) receptor, and delta‐opioid receptor agonists. The impact of SSRI (selective serotonin receptor inhibitors) relies on the intact synthesis of 5‐HT as 5‐HT (5‐hydroxytryptamine) complies with the efficacy of antidepressant effect on the swimming inspection (Cryan et al., 2005). It is also assumed that the existence of phytochemicals in the MEPN improves mobility in the FST and TST by upraising the transmission of 5HT. The psychological impacts of the GABA transmitter and nitric oxide synthase during the FST were blocked by the scarcity of 5‐HT synthesis which ascertains that augmented transmission of 5‐HT is associated with their antidepressant activity.

The number and time period of exploratory head dips were measured in HBT with the dose‐dependent administration of MEPN (100, 200, and 400 mg/kg b.w.; p.o.). This method is advantageous due to the analytical versatility of HBT, and different behavioral reactions can be easily detected and evaluated when subjected to unfamiliar circumstances. The animals' head‐dipping tendency is directly linked to their psychological condition (Ali et al., 2015). This result is consistent with the fact that after the administration of nonsedative doses of diazepam, the intensity of head dipping and early latency of the first head dip were observed in the hole‐board. However, with enhanced head dipping and early head dipping, anxiolytic status has been defined. This suggests that the animal's distress can be demonstrated by a decline in head‐dipping activity in the hole‐board experiment (Takeda et al., 1998). By suppressing an enzyme that breaks down monoamines (norepinephrine, serotonin, and dopamine) and preventing reuptake in the synaptic cleft, some first‐line anxiolytic and antidepressant drugs improve monoamine function. First‐line medications are often self‐prescribed as anxiolytic and/or antidepressant drugs by the patient himself with or without a prescription. Benzodiazepines that amplify GABAergic inhibitory transmission are the most commonly utilized anxiolytic agents (Tatarczyńska et al., 2001). Few anxiolytic drugs have activities that may interact with antidepressant actions in pathophysiology. Serotonin agonists such as buspirone and gepirone are expected to work on 5‐hydroxytryptamine‐1A (5‐HT1A) presynaptic and postsynaptic receptors and are used as both antidepressants and as anxiolytic moieties (Schechter et al., 2005).

The study revealed a significant insight on the antipyretic study of MEPN utilizing biological and computational studies of the selected compounds of *P. nigrum*. The possible mechanism of action of antipyretic agents might be attributed by inhibition of prostaglandin synthesis, that is, cessation of synthesis of prostaglandin by blocking cyclooxygenase by paracetamol (Rudra et al., 2020). Inhibition of several pyrexia‐inducing mediators is accountable for the antipyretic effect (Muhammad et al., 2012). Therefore, it can be predicted that, MEPN might possess the antipyretic activity due to the presence of bioactive phytochemicals by inhibiting the prostaglandin synthesis like other analgesic drugs, as both of those reduced the rectal temperature of yeast injected mice. In case of nonsteroidal anti‐inflammatory drugs (NSAIDS), it is also established that they can show antipyretic activity through the inhibition of prostaglandin synthesis within the hypothalamus (Vane & Botting, 1998).

Findings of thrombolytic research indicate that during the thrombolytic property evaluation, the coagulation procedure is divided into three stages: prothrombin activator formation, thrombin training, and fibrin production. Antithrombotic or thrombolytic agents can interrupt the formation of a thrombus. The core part of the thrombolytic mechanism is to eliminate plasmin fibrin, which can be triggered by inactive plasminogen activators. Streptokinase (SK) and urokinase (UK) work through the indirect clot lysis route. The thrombolytic enzymes effectively eliminate the fibrin in the indirect mechanism which was also proved by in vitro thrombolysis studies (Yuan et al., 2012). This research has evidently shown that MEPN is capable of inhibiting coagulation which can be an integral component of thrombolytic treatment management that does not impair the usual clotting process in the body low‐dose pattern. Urokinase (UK) and streptokinase (SK) lysis the clot indirectly. The thrombolytic enzymes dissolve directly to the fibrin in the indirect mechanism (Yuan et al., 2012). This research has already explored that MEPN can suppress coagulation and does not impact the normal body coagulation process at a low dose. Therefore, low dose of MEPN has been recommended for blood clot mitigation.

Molecular docking studies have been widely employed to predict ligand–target interactions and to intensify the understanding of natural products' bio‐activities. It also offers additional insights regarding possible binding mechanisms within the protein binding pockets (Khan et al., 2019). To understand the implications of this fact, the biological studies have been explained and validated through the molecular docking study. Seven typical compounds of *P.nigrum* have been chosen to comply the docking study to get a deeper examination following biological characteristics (anxiolytic, antidepressant, antipyretic, and thrombolytic). These compounds were then docked against three targets, namely the potassium channel (PDB ID: 4UUJ), human serotonin transporter (PDB ID: 5I6X), m‐PGES 1 (PDB ID: 4YK5), and tissue plasminogen activator (PDB ID: 1A5H) for anxiolytic, antidepressant, antipyretic, and thrombolytic studies correspondingly. Human serotonin transporters (PDB ID: 5I6X) react to the ligands via a series of bonds which result in docking values of −3.08 to −7.14 kcal/mol. These results indicate that these phytoconstituents are substantially responsible for antidepressant activity through the interaction with these target proteins. Molecular docking of the selected phytoconstituents with the potassium channel (PDB ID: 4UUJ) also employed to evaluate the anxiolytic docking study, and the docking score has been documented ranging from −1.0 to −7.9 kcal/mol. Subsequently, the docking score for the antipyretic study has been found ranging from −4.5 to −6.9 kcal/mol. Among all the compounds, cepharadion A, piperolactam D, paprazine, and piperine have shown the significant docking score against plasminogen tissue activator (PDB ID: 1A5H) in the thrombolytics docking study and the highest score against tissue plasminogen activator enzymes has been seen in Cepharadione A. This research suggests that the bioactive compounds (cepharadione A, paprazine, piperine, 10‐tricosanone, and piperolactam D) may be responsible for the thrombolytic activity of the MEPN.

Regarding the findings of molecular docking analyses against the human serotonin transportation, potassium channel, m‐PGES 1, and tissue plasminogen activator, the pharmacokinetics and toxicological parameters of the compounds have been checked. All the compounds obeyed Lipiniski's drug‐likeliness rules. Therefore, these interpretations of identified substances are incredibly supportive to establishing a new pharmaceutical agent (Lipinski, 2004; Rudra et al., 2020; Zhang & Wilkinson, 2007).

The findings suggest that the compounds meet Lipinski's rules, which indicate that they might act as effective drug candidates, and that they are risk free for the oral use (Table 3). Furthermore, we used the admetSAR online tool to evaluate the toxicological parameters of the assigned plant compounds as drug safety is an essential factor for making it a good medicinal product (Cheng et al., 2012).

PASS prediction is a computational tool that simulates the constituents depending on varying degrees of bioactivity and clarifies multiple effectivities of the components (Poroikov & Filimonov, 2005). This research explained compounds with a greater value of Pa (trustworthy activity) than Pi (trustworthy inactivity) have been considered acceptable for a certain biological activity. The comprehensive analysis therefore revealed noteworthy findings regarding the selected compounds. The results might become attributable due to the combined orientation of many phytochemicals, including established and nonreported phytochemicals.

## 5 CONCLUSION

The interpretation of the biological results of this scientific research reflects that the aerial part of *Piper nigrum* can be a prominent source of antidepressant, anxiolytic, antipyretic, and clot‐lytic agents. Besides, the molecular docking analysis of the bioactive phytoconstituents displayed promising binding affinity to the specific proteins and the ADME/T study showed their drug‐like characters. The experimental findings have been consistent with PASS predictions for bioactive constituents too. Thus, the computational study has validated the experimental results of biological activities and provided a promising insight for considering *P. nigrum* as a notable drug candidate. Additional studies are also recommended as it is suggested that structural alteration of these compounds may show improved docking score and better therapeutic importance.

## CONFLICT OF INTEREST

The authors declare that they have no competing interest.

## AUTHOR CONTRIBUTION

U.E. and S.R. conceptualized the study. U.E., S.A., A.P., and S.R.R. performed data curation. N.U.E., S.R., and S.A. performed formal analysis. N.U.E., S.R., U.K., S.R.R., and A.P. investigated the study. S.R., N.U.E., and S.M. M.H. designed methodology. S.M.M.H. and N.U.E. administered the project. S.M., M.H. and A.G. provided resources. N.U.E. implemented software programs. S.A. and S.M., M.H. validated the data. N.U.E., S.R., U.K., and S.A. performed visualization. S.R, N.U.E., and S.A. wrote the original draft. S.A., N.U.E., and A.G. reviewed and edited the manuscript. A.G. and S.R. supervised the study.

## ETHICAL APPROVAL

In compliance with the ethical guidelines laid out in the 2013, named Helsinki Declaration 2013, all biological activity screenings have been completed. Tests were conducted and processed according to the guidelines of animal euthanasia in 2013 version and were euthanized according to the Swiss Academy of Medical Sciences and the Swiss Academy of Science.

## Data Availability

Google Scholar (https://scholar.google.com/) and PubMed (https://www.ncbi.nlm.nih.gov/pmc/about/intro/) have been used to search available articles in order to write the introduction and discussion part of the manuscript. In addition, PubChem (https://pubchem.ncbi.nlm.nih.gov/) and rcsbPDB (https://www.rcsb.org/search) websites have also been used to collect the structures of the ligands and proteins. Besides, admeSAR (http://lmmd.ecust.edu.cn/admetsar2/) was used to gather the information of ADME/T properties so that it can be included in in silico investigations.
